# Increase in podocyte number induced by SV40T antigen during the fetal stage does not expand glomerular size in mice

**DOI:** 10.14814/phy2.71114

**Published:** 2026-10-02

**Authors:** Taiji Matsusaka, Koichiro Ichimura, Takayuki Miyaki, Masahiro Koizumi

**Affiliations:** ^1^ Institute of Medical Science Tokai University School of Medicine Isehara Kanagawa Japan; ^2^ Department of Physiology Tokai University School of Medicine Isehara Kanagawa Japan; ^3^ Department of Internal Medicine, Division of Nephrology, Endocrinology, and Metabolism Tokai University School of Medicine Isehara Kanagawa Japan; ^4^ Department of Anatomy and Life Structure Juntendo University Graduate School of Medicine Tokyo Japan

**Keywords:** glomerulosclerosis, oligomeganephronia, podocyte, Simian virus 40 large T antigen, transgenic mouse

## Abstract

In healthy kidneys without injury or compensatory hypertrophy, glomerular volume generally correlates with the podocyte number. In this study, we investigated whether experimentally increasing podocyte numbers can expand glomerular volume. We generated transgenic mice in which the SV40 large T antigen (SV40T) was selectively expressed in podocytes in a doxycycline‐inducible manner. In adult mice, doxycycline treatment successfully induced cell cycle re‐entry in podocytes, albeit with low efficiency. However, prolonged treatment caused proteinuria and podocyte injury. When doxycycline was administered during embryogenesis, newborn mice clearly exhibited an increase in podocyte proliferation, leading to an average of 54% increase in podocyte number. These additional podocytes frequently formed multiple layers and detached into Bowman's space or tubular lumen; consequently, the glomerular tuft area did not increase. By postnatal Day 7, excess podocytes disappeared, and podocyte counts returned to levels comparable to those in control mice. These findings indicate that glomerular volume is determined at or before the S‐shaped body stage, and that forced increases in podocyte number induced by SV40T at later developmental stages are insufficient to expand glomerular volume.

## Graphical abstract

Introduction of SV40 large T antigen (SV40T) in embryonic mouse podocytes via a Tet‐On system markedly increased podocyte number at birth (P0). However, the supernumerary podocytes formed multilayers and detached into the urinary space, failing to enlarge the glomerular tuft area. By postnatal day 7 (P7), the excess podocytes were lost, and podocyte density normalized. These results indicate that forced podocyte proliferation after the S‐shaped body stage cannot override the structural constraints that define glomerular volume.

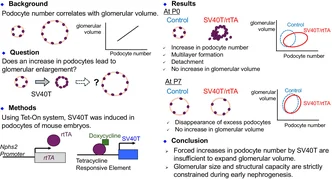

## 1 INTRODUCTION

Podocytes are terminally differentiated cells that function as critical components of the glomerular filtration barrier. Under normal physiological conditions, podocytes do not proliferate after the S‐shape stage of nephron development (Schell et al., 2014). When a substantial number of nephrons are lost by various kidney diseases, the remaining glomeruli undergo compensatory hypertrophy. Beyond the adaptation limit, podocytes are lost, which causes glomerulosclerosis (Fukuda et al., 2012). Thus, the limited proliferative capacity of podocytes is a major factor in the irreversible progression of chronic kidney disease across various glomerular disorders.

While aging and various pathological conditions reduce podocyte number, glomerular volume increases during normal growth or compensatory hypertrophy following nephron loss. Podocyte number and glomerular volume generally correlate in the absence of these confounding factors (Haruhara et al., 2022; Puelles et al., 2015).

Podocytes are central to glomerular morphogenesis during embryonic development. Cells adjacent to the cleft of the S‐shaped body differentiate into podocytes, which secrete vascular endothelial growth factor to recruit endothelial precursor cells. Endothelial cells subsequently secrete PDGF‐B to attract mesangial cells. Furthermore, podocytes and glomerular endothelial cells produce components of the glomerular basement membrane (GBM), including laminin and collagen IV. Notably, the mature collagen IV isoform (α3α4α5) is exclusively produced by podocytes (Schell et al., 2014).

These developmental observations suggest that the podocyte number may be a primary determinant of glomerular volume. However, whether experimentally expanding the podocyte population can increase the glomerular size remains an open question. To address this question, we utilized the Tet‐On system to generate transgenic mice, allowing for podocyte‐specific, doxycycline‐inducible expression of the Simian virus 40 large T antigen (SV40T). SV40T promotes cell‐cycle progression primarily by inhibiting the tumor suppressor proteins p53 and retinoblastoma protein (Rb) and is commonly used to immortalize various cell types in culture, including podocytes (Sullivan & Pipas, 2002). Using this model, we tested whether expanding the podocyte population in vivo is sufficient to increase glomerular size.

## 2 MATERIALS AND METHODS

### 2.1 General procedure of animal experiments

All animal experiments were approved by the Animal Experimentation Committee of Tokai University School of Medicine (approval numbers 122034, 133015, 144028, and 155027) and were conducted in accordance with the Guide for the Care and Use of Laboratory Animals (U.S. National Institutes of Health) and the ARRIVE guidelines (https://arriveguidelines.org). Mice were housed in groups of five per cage in a temperature‐controlled environment (23°C) with a 12‐h light/dark cycle. The animals had access to standard laboratory chow (Clea Japan, CE‐2) and sterile water ad libitum. Specific pathogen‐free conditions were maintained throughout the study. For all mice, physical appearance and activity were checked five times per week. The mice were humanely euthanized under isoflurane inhalation anesthesia.

### 2.2 Generation and establishment of the TRE‐SV40T transgenic mouse line

A 2.7‐kb BamHI–BglI fragment of SV40 genomic DNA encoding the T antigen was subcloned into the pBI‐G Tet vector (Clontech) at the NotI and SalI sites. A 7.9‐kb SalI–SapI fragment was excised and used as the transgene. The TRE‐SV40T construct contains a bidirectional promoter (Pbi‐1) that drives the expression of the SV40T and *lacZ* reporter genes. The Pbi‐1 promoter contains a tetracycline‐responsive element (TRE) that is activated by the reverse tetracycline‐responsive transcription activator (rtTA) in the presence of doxycycline.

TRE‐SV40T DNA was microinjected into 200 fertilized eggs obtained from D2C6F1 × C57BL/6 mating. Seven founder mice were generated, and six transgenic lines were established by mating with wild‐type C57BL/6 mice. These lines were then crossed with Nphs2‐rtTA mice, provided by Dr. J.B. Kopp (Shigehara et al., 2003). Nphs2‐rtTA mice express rtTA specifically in the podocytes. Nphs2‐rtTA mice were generated on the FVB/N background. Both lines were backcrossed with C57BL/6 mice three times before cross‐mating.

One SV40T line crossed with the rtTA line showed selective doxycycline‐inducible podocyte expression of transgenes. SV40T+/rtTA+ mice were subsequently backcrossed with C57BL/6 mice three times and then crossed with each other.

Bcl2 knockout (KO) mice (B6.129P2‐Bcl2<tm1Tsu>/TsuRbrc, RBRC01593) were obtained from RIKEN BioResource Research Center (Kamada et al., 1995). They were mated with the SV40T/rtTA line.

Genotyping was performed by PCR using genomic DNA extracted from tail biopsies with the primers listed in Table S1.

To induce SV40T expression in podocytes, TRE‐SV40T/Nphs2‐rtTA (SV40T+/rtTA+) mice were treated with doxycycline (2 mg/mL in drinking water containing 5% sucrose). Control mice received 5% sucrose alone.

### 2.3 Experiments in adult mice

A total of 128 SV40T+/rtTA+ and control mice were treated with doxycycline for 3–42 days. In some mice, BrdU was continuously infused using a mini‐osmotic pump (Alzet 1007 or 2002) to label proliferating cells. Specifically, female mice were selected for the experiments involving urinary albumin‐to‐creatinine ratio (U‐ACR) measurements (Figures S4 and S5) because female C57BL/6 mice exhibit a lower and more stable baseline U‐ACR compared to males, allowing clearer detection of subtle therapeutic or toxic effects. For all other experiments evaluating cellular proliferation and histology, both male and female mice were utilized.

In the experiment for measuring U‐ACR (Experiment for Figure S4), female SV40T+/rtTA+ and SV40+/rtTA‐ mice (12–13 weeks of age) (*n* = 6 per group) were treated with doxycycline (2 mg/L) and losartan (0.5 g/L) in drinking water containing 5% sucrose for 4 days (from Days 1 to 4), followed by losartan alone for 8 days (from Days 5 to 12). The same number of female SV40T+/rtTA+ and SV40T+/rtTA‐ mice were treated with doxycycline alone from Day 1 to 4. Twenty‐four‐hour urine samples were collected using metabolic cages on Day 0, 1, 4, 5, and 12. Urinary creatinine concentrations were measured using an automated analyzer (SRL, Tokyo, Japan), and albumin concentrations were determined using an ELISA kit (Albuwell, Exocell, Philadelphia, PA, USA).

To examine the effects of continuous doxycycline treatment (Experiment for Figure S3), adult SV40T+/rtTA+ mice (8–25 weeks of age) were continuously treated with doxycycline for 4, 7, 14, 21, 28, or 42 days, and histologically examined. The number of mice (male mice) was 5 (5) for no treatment, 4 (3) for 4‐day treatment, 6 (5) for 7 days, 5 (2) for 14 days, 4 (4) for 21 days, 5 (5) for 28 days, and 3 (0) for 42 days. Both male and female mice were analyzed.

In the experiment for increasing podocyte numbers in adult mice (Experiment for Figure S4), 11 female SV40T+/rtTA+ mice and 12 female SV40T+/rtTA‐ mice (9–22 weeks of age) were treated with doxycycline and losartan for 4 days, followed by losartan alone for 10 days. This cycle was repeated six times.

### 2.4 Experiments in newborn mice (P0 and P7)

Mice carrying TRE‐SV40T, Nphs2‐rtTA, and mutant Bcl2 alleles were crossed to obtain SV40T+/rtTA+/Bcl2^−^/^−^ mice. Matings were arranged to avoid the generation of homozygous TRE‐SV40T or Nphs2‐rtTA mice. This mating was set aiming to obtain at least 5 mice in each genotype. Pregnant females were treated with doxycycline in their drinking water from embryonic Day 14.5 (E14.5) until delivery or postnatal Day 7 (P7).

A total of 105 live newborn mice were obtained by natural delivery (38 pups) or cesarean section (67 pups) from 17 mothers and were genotyped (Table S2). Five pups died at delivery, three of which were Bcl2^−^/^−^. An additional seven mothers were allowed to nurse their litters while continuing the doxycycline treatment. Twenty‐six pups were analyzed at P7. Both male and female mice (P0 and P7) were analyzed.

### 2.5 Histological analysis

A minimum of 10 serial or semi‐serial paraffin sections (2 μm thick) were prepared and evaluated per biological sample, and Periodic acid‐Schiff (PAS) staining and immunostaining were performed. Antibodies and staining conditions are listed in Table 1.

**TABLE 1:** Antibodies and a kit used for the study.

Antigen	Antibody or kit	Company	Catalogue number	RRID	Reference	Pretreatment	Dilution	Enhancement	
SV40T	Rabbit polyclonal anti‐SV40T	Santa Cruz Biotechnology	sc‐20800	AB_661493	https://doi.org/10.3748/wjg.v12.i17.2785	Heat	1/500	CanGet Signal solution	Figures 1 and 2
Synaptopodin	Mouse monoclonal anti‐synaptopodin	Progen	#65194	AB_2909601	https://doi.org/10.34067/KID.0000001034	Heat	1/100		Figure 1, Figure S5
Beta galactosidase (lacZ)	Mouse monoclonal anti‐beta galactosidase	Promega	Z3781	AB_430877	https://doi.org/10.1523/JNEUROSCI.3109‐11.2011	Heat	1/1000		Figure 1
Ki67	Mouse monoclonal anti‐Ki67	Novocastra	NCL‐Ki67‐MM1	AB_442101	https://doi.org/10.1034/j.1600‐0463.2003.1110505.x	Heat	1/100		Figures 1 and 2
p57	Goat polyclonal anti‐p57Kip2	Santa Cruz Biotechnology	sc‐1039	AB_2078158	https://doi.org/10.1152/ajprenal.00414.2019	Heat	1/100		Figure 1
Podocin	Rabbit polyclonal anti‐podocin	Sigma‐Aldorich	P0372	AB_261982	https://doi.org/10.1186/s12882‐023‐03420‐x	Trypsin	1/1000		Figures 1 and 2
Podocin	Rabbit polyclonal anti‐podocin	Sigma‐Aldorich	P0372	AB_261982	https://doi.org/10.1186/s12882‐023‐03420‐x	Pepsin	1/5000	CanGet Signal solution	
Bromodeoxyuridine	BrdU staining kit	Oncogene Research Products (now a part of EMD Millipore)	#HSC24		https://doi.org/10.1681/ASN.2004080720	Trypsin			Figure 1, Figure S1
Nephrin	Guinea pig polyclonal anti mouse nephrin	Progen	GP‐N2	AB_1542487	https://doi.org/10.1152/ajprenal.00412.2023	Heat	1/100		Figure 2
Desmin	Mouse monoclonal anti‐desmin	DAKO	M0760	AB_2335684	https://doi.org/10.1007/s00428‐005‐0134‐9	Heat	1/50		Figure 2
WT‐1	Rabbit polyclonal anti‐WT‐1	Santa Cruz Biotechnology	sc‐192	AB_632611	https://doi.org/10.1038/nm.3142	Heat	1/100		Figure S5
WT‐1	Anti‐Wilms Tumor Protein antibody [CAN‐R9(IHC)‐56‐2]	Abcam	ab89901	AB_2043201	https://doi.org/10.1038/nm.2550	Heat	1/4000		Figure 3
Aurora B	Rabbit polyclonal anti‐AuroraB	abcam	ab2254	AB_302923		Heat	1/250	CanGet Signal solution	Figure S2
Cleaved Lamin A	Cleaved Lamin A (Small Subunit) Antibody	Cell Signaling Technology	#2035	AB_2234647	https://doi.org/10.1016/j.ymeth.2007.11.004	Heat	1/100	CanGet Signal solution	Figure S6
gammaH2AX	Phospho‐Histone H2A.X (Ser139) (20E3) Rabbit Monoclonal Antibody	Cell Signaling Technology	#9718	AB_2118009	https://doi.org/10.1016/j.cmet.2023.10.014	Heat	1/1000	CanGet Signal solution	Figure S6
Podocalyxin	Rat anti mouse podocalyxin antibody	R&D systems	MAB1556	AB_2166010	https://doi.org/10.1038/s41598‐020‐64907‐3	Heat	1/100	CanGet Signal solution	Figure 3, Figure S6
Rabbit IgG	Goat Anti‐Rabbit IgG Antibody (H+L), Biotinylated	Vector	BA‐1000	AB_2313606			1/200		
Goat IgG	Rabbit Anti‐Goat IgG Antibody (H+L), Biotinylated	Vector	BA‐5000	AB_2336126			1/200		
Mouse IgG	M.O.M. Immunodetection Kit	Vector	BMK‐2202	AB_2336833					
Rat IgG	SignalStain Boost IHC Detection Reagent (AP, Rat)	Cell Signaling Technology	#15764						

For double immunostaining (synaptopodin + SV40T, Ki67, or WT1), paraffin sections were first stained for synaptopodin using the ABC method (Vector PK‐7100), developed with diaminobenzidine (DAB), washed in 0.1 M glycine buffer (pH 2.2), and then stained for the second marker using Ni/Co‐DAB.

For double staining (podocalyxin + SV40T, Ki67, WT1, γH2AX, or cleaved Lamin A), sections were first stained for SV40T, Ki67, WT1, γH2AX, or cleaved Lamin A using the ABC method and DAB development, washed in 0.1 M glycine buffer, and then stained for podocalyxin using an alkaline phosphatase‐conjugated secondary antibody and substrate (CST #15764, #76713).

For podocin + BrdU double staining, paraffin sections were pretreated with trypsin, incubated simultaneously with anti‐BrdU and anti‐podocin antibodies, and visualized using a Cy3‐anti‐rabbit antibody and fluorescent avidin.

Other immunohistochemical staining was performed using biotin‐conjugated secondary antibodies, ABC kit (PK7100, Vector), and DAB.

### 2.6 Semi‐quantification of glomerulosclerosis and podocyte injury in adult mice

Glomerulosclerosis was scored on PAS‐stained 2‐μm sections using a scale of 0–4, where 0 indicates no lesion and 1–4 represent ≤25%, 26%–50%, 51%–75%, and >75% involvement of the glomerular tuft, respectively.

Podocin and nephrin staining of paraffin sections was graded as 0 (no staining), 1 (≤25% of tuft area), 2 (26%–50%), 3 (51%–75%), or 4 (>75%). The average score per mouse was then calculated. Desmin expression was used to identify the injured podocytes and mesangial cells. Desmin‐stained sections were counterstained with PAS. The percentage of glomeruli containing desmin‐positive podocytes was recorded. Inner podocytes could not always be distinguished from mesangial cells and were therefore not evaluated.

For these analyses, all non‐tangential glomeruli visible within each section were evaluated under a 40× objective lens (averaging 92 glomeruli per mouse, range: 62–179).

### 2.7 Morphometric analysis

In adult mice, podocyte number and glomerular area were measured on 2‐μm paraffin sections doubly stained for WT1 and synaptopodin to increase the specificity of podocyte staining. Samples from each experimental set were embedded in a single paraffin block to standardize section thickness. Cells that were double‐positive for WT1 and synaptopodin were counted in all glomeruli per kidney section. The glomerular area was measured using AxioVision 2.05 software. Glomeruli <1500 μm^2^ were considered tangential and were excluded. The analyses were performed blindly to the doxycycline treatment. The mean podocyte number was calculated from more than 60 non‐tangential glomeruli per mouse.

In SV40T+/rtTA+ mice at postnatal day (P) 0, podocytes frequently formed clusters protruding into the tubular lumens or were dispersed in Bowman's space, making the outer glomerular boundary difficult to define. Therefore, the glomerular tuft area, rather than the total glomerular area, was measured in the newborn mice. WT1‐stained paraffin sections were imaged using a BX63 microscope (Evident), and entire kidney cross‐sections were digitally stitched using the cellSENS software (Evident). To avoid sampling bias, all non‐tangential glomeruli in the inner cortex across the entire section from all SV40T−/rtTA‐ and SV40T+/rtTA+ mice were individually examined at full‐screen magnification (raw data in Table S3). For each glomerulus, the inner boundary of the WT1‐positive podocytes attached to the GBM was traced manually to measure the tuft area. The number of WT1‐positive podocytes was counted in each tuft. Parietal epithelial cells, which stained less intensely for WT1, were excluded. Mean number of WT1‐positive podocytes per glomerulus and the mean tuft area were calculated for each mouse. At P0, the following numbers of mice were analyzed: eight (3 males) SV40T−/rtTA‐/Bcl2^+/+^, five (2) SV40T+/rtTA+/Bcl2^+/+^, nine (3) SV40T−/rtTA‐/Bcl2^−/−^, and four (3) SV40T+/rtTA+/Bcl2^−/−^.

For mice at P7, all glomeruli in the inner half of the cortex were similarly analyzed. Because the sample size was insufficient for separate statistical analysis, Bcl2^+/+^ and Bcl2^+/−^ mice were combined into a single group. Eight SV40T−/rtTA‐ (five SV40T−/rtTA‐/Bcl2^+/+^, three SV40T−/rtTA‐/Bcl2^+/−^) and five SV40T+/+ (two SV40T+/rtTA+/Bcl2^+/+^, three SV40T+/rtTA+/Bcl2^+/−^) mice were compared.

### 2.8 Immuno‐electron microscopy

For electron microscopy (EM) and immuno‐EM, the kidneys were perfused with phosphate‐buffered saline (PBS), followed by 4% paraformaldehyde in PBS. Kidneys were fixed overnight at 4°C in 4% paraformaldehyde in 50 mM phosphate buffer (pH 7.2), cryoprotected in graded 5%–20% sucrose/PBS solutions, embedded in OCT compound, and frozen on acetone/dry ice. Sections (8 μm) were cut, air‐dried, fixed in 4% paraformaldehyde, rinsed with PBS, incubated in 0.114% periodic acid for 10 min, then in 3 mM NaBH₄ for 30 min, and rinsed again. BrdU staining was performed using a BrdU staining kit (Oncogene, HCS24), and signals were visualized using avidin‐peroxidase and DAB. The tissues were post‐fixed in osmium tetroxide, dehydrated, and embedded in EPON resin (NisshinnEM, Quetol‐812, 341‐1).

### 2.9 Statistical analysis

The U‐ACR was log‐transformed before analysis using a linear mixed‐effects model. Semiquantitative indices of podocyte dysregulation in adult SV40T+/rtTA+ mice were analyzed using the Kruskal–Wallis test and Steel's test. Podocyte number and glomerular area in adult mice were analyzed using the Mann–Whitney *U* test. In newborn mice (P0), podocyte number and glomerular tuft area were compared using the Kruskal–Wallis rank‐sum test with Steel‐Dwass post hoc analysis. At P7, data were analyzed using the Mann–Whitney *U* test. A *p* value <0.05 was considered statistically significant. Statistical analyses were performed using EZR.

### 2.10 Usage of artificial intelligence

ChatGPT‐4 (assessed December 2025), Gemini 3 Flash (February 2026), and Gemini 3.5 Flash (July 2026) were used to assist in grammar and spelling correction throughout the manuscript and to refine the logical flow of the Discussion section. All AI‐assisted text suggestions were critically reviewed and revised by the authors to guarantee accuracy, scientific precision, and integrity of the content.

## 3 RESULTS

### 3.1 Podocytes re‐entered the cell cycle in doxycycline‐treated adult SV40T+/rtTA+ mice

The SV40T+/rtTA+ mice were treated with doxycycline. Immunostaining confirmed the selective expression of both SV40T and lacZ in podocytes after 4–14 days of doxycycline administration. Approximately 20%–30% of podocytes (2–3 per glomerular section) expressed SV40T. In these mice, 9.3%–17.7% of podocytes (approximately 1–2 per glomerulus) were positive for Ki67, a marker of late G1 through M phases (Figure 1; Table S4). In addition, SV40T‐expressing podocytes incorporated bromodeoxyuridine (BrdU), indicating S‐phase entry (Figure 1; Figure S1). These podocytes also expressed Aurora kinase B (Aurora B), a marker of S through early G1 phases, and occasionally exhibited mitotic morphology (Figure S2). Conversely, the cyclin‐dependent kinase inhibitor, p57^Kip2^, which is typically expressed in mature podocytes, was downregulated in SV40T‐expressing cells (Figure 1). Together, these findings demonstrated that SV40T forced podocytes to re‐enter the cell cycle. Podocytes frequently became multinucleated or developed enlarged nuclei, suggesting cytokinesis failure.

**FIGURE 1:**
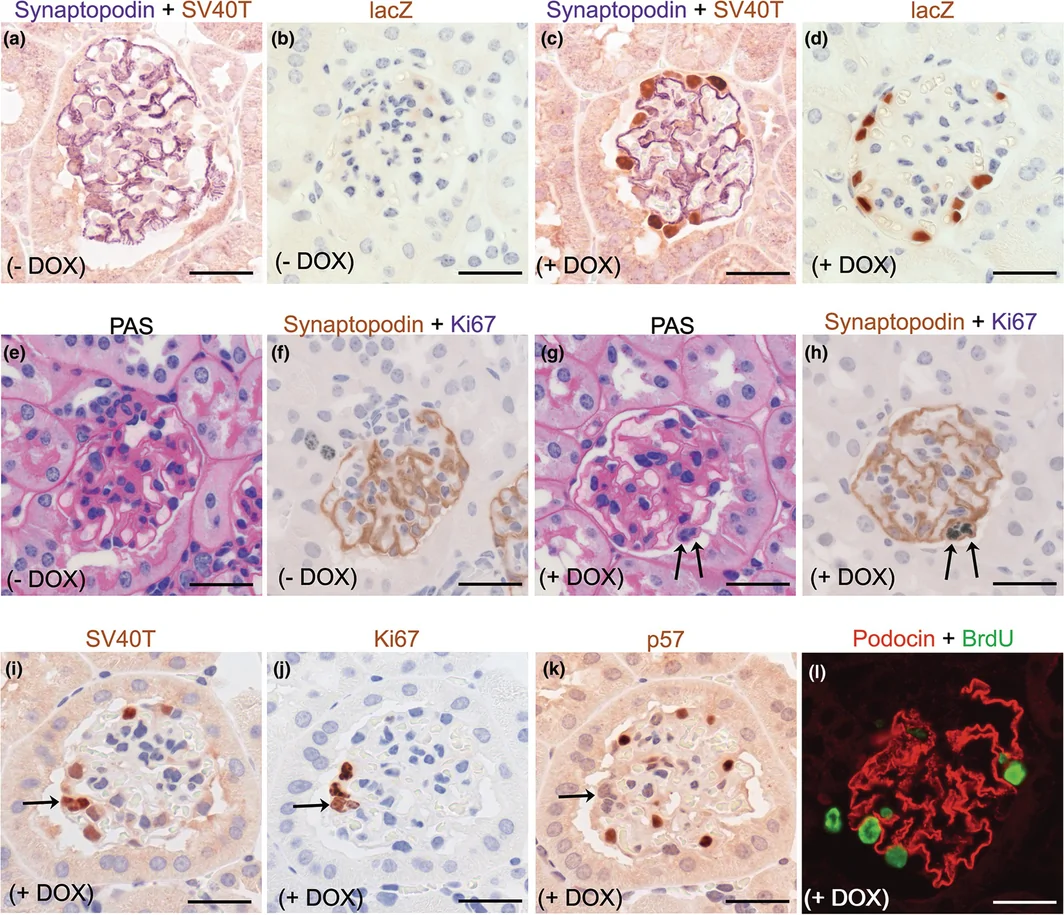
Podocyte‐selective and doxycycline‐inducible SV40T expression and cell‐cycle re‐entry in adult SV40T+/rtTA+ mice. SV40T and lacZ proteins are expressed in podocytes (identified by synaptopodin staining) of SV40T+/rtTA+ mice treated with doxycycline (+DOX) (c, d), but not in untreated mice (‐DOX) (a, b). Ki67 is detected in podocytes of doxycycline‐treated SV40T+/rtTA+ mice (arrows, h) but not in untreated mice (f). Panels (e) and (g) show PAS staining of sections adjacent to (f) and (h), respectively. Ki67‐positive podocytes are binuclear but retain normal morphology and synaptopodin expression. Ki67 staining (j) is observed in a subset of SV40T‐positive podocytes (i). p57Kip2 (k), normally expressed in podocytes, is suppressed in Ki67‐positive podocytes (arrow). Panels (i), (j), and (k) are serial sections. When SV40T+/rtTA+ mice were infused with bromodeoxyuridine (BrdU) during doxycycline treatment, BrdU‐positive nuclei were frequently detected in podocytes, identified by their location external to podocin staining (l). Bars represent 20 μm.

None of these abnormalities were observed in wild‐type mice, single‐transgenic controls treated with doxycycline, or SV40T+/rtTA+ mice not exposed to doxycycline.

### 3.2 Podocyte injury in doxycycline‐treated adult SV40T+/rtTA+ mice

Doxycycline treatment induced albuminuria in SV40T+/rtTA+ mice. The average U‐ACR was 15.76 ± 16.44 at Day 7 and 40.27 ± 39.46 at Day 14, whereas control mice maintained ratios of 0.02 ± 0.01 and 0.01 ± 0.00 on Days 7 and 14, respectively. Kidney morphology remained largely preserved for the first week of doxycycline treatment (Figure 2), aside from the microvillous transformation of podocytes (Figure S1). However, after 2 weeks, nephrin and podocin expression was reduced, and desmin was upregulated in SV40T+/rtTA+ mice. After four or more weeks of treatment, all SV40T+/rtTA+ mice developed focal segmental glomerulosclerosis (FSGS) of the usual type (Figure 2; Figure S3). Neither crescent, pseudocrescent, nor collapse was observed. Similar to other FSGS models, SV40T+/rtTA+ mice with severe glomerular damage exhibited tubule‐interstitial damage. Notably, damaged podocytes did not express SV40T, likely because Nphs2 promoter‐driven rtTA expression is lost during injury. These results indicated that podocyte proliferation cannot be sustained in adult mice without triggering injury, which ultimately suppresses transgene expression. Albuminuria induced by 4 days of treatment was mild, attenuated by losartan, and resolved after doxycycline withdrawal (Figure S4). Therefore, we tested multiple intermittent short doxycycline treatments in combination with losartan to promote podocyte expansion. After six treatment cycles, the podocyte counts per section increased slightly, but the effect was modest (Figure S5).

**FIGURE 2:**
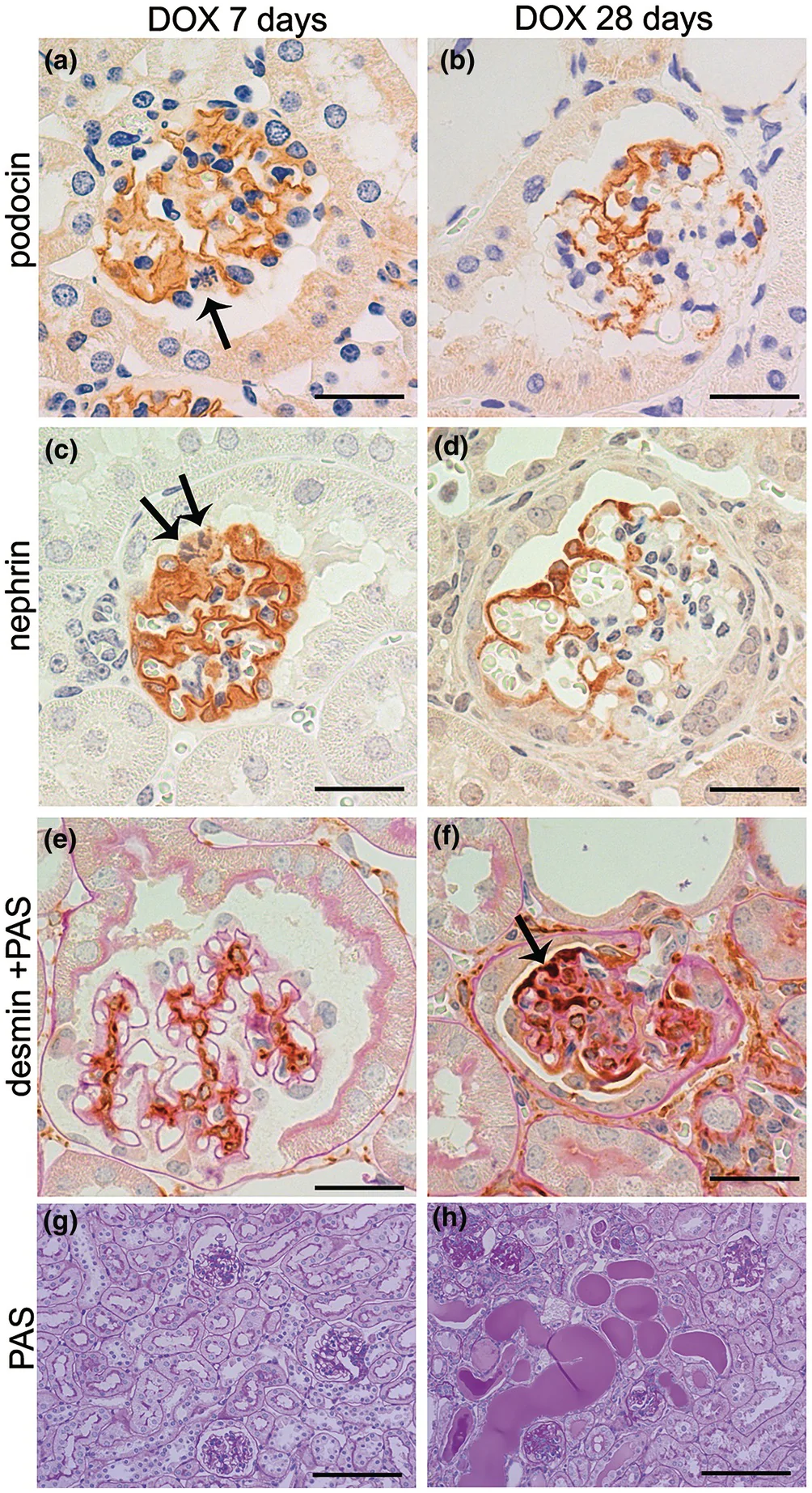
Dysregulation of podocytes in adult doxycycline‐treated SV40T+/rtTA+ mice. After 7 days of doxycycline treatment, podocin (a) and nephrin (c) show preserved expression and desmin (e) is restricted to mesangial cells. Arrows in (a) and (c) indicate condensed chromosomes. Glomerular and tubular morphology are normal (g). At 28 days of treatment, segmental glomerulosclerosis is frequently observed, accompanied by reduced podocin (b) and nephrin (d) expression and marked desmin upregulation in the glomerular segments (f, arrow). Kidneys with severe glomerulosclerosis show tubular damage (h). Bars represent 20 μm.

### 3.3 Embryonic podocyte proliferation and glomerular volume

We next attempted to induce podocyte proliferation during embryogenesis, aiming to increase podocyte numbers in neonatal mice and to assess the impact of podocyte proliferation on oligomeganephronia. For this purpose, we crossed SV40T, rtTA, and Bcl2 KO lines. Homozygous Bcl2‐deficient mice (Bcl2^−/−^) are a model of oligomeganephronia characterized by a profound reduction in nephron number and glomerular hypertrophy (Gassler et al., 1998; Nagata et al., 1996). Pregnant dams were treated with doxycycline from embryonic day (E) 14.5 to delivery.

Sixty‐seven pups were obtained from 11 dams via cesarean section, and 38 pups were from 6 dams via natural delivery (Tables S2 and S3). As previously reported (Nagata et al., 1996), Bcl2^+/−^ mice exhibited a normal nephron architecture that was indistinguishable from that of Bcl2^+/+^ mice. Podocytes were markedly increased in SV40T+/rtTA+ mice (regardless of the Bcl2 background) (Figure 3). Nearly all podocytes in the inner cortex expressed SV40T and most were positive for Ki67 (Figure 3h–j). However, podocytes frequently formed multilayers or clusters, which occasionally protruded into the tubular lumen. Most glomerular precursors in the outer renal cortex had not yet expressed podocalyxin or SV40T. In rare and slightly more mature superficial glomeruli, podocalyxin was expressed, but SV40T expression was sporadic, and these podocytes neither expressed Ki67 nor formed multilayers (Figure S6). Unlike cells in crescents or pseudocrescents, the podocytes in SV40T+/rtTA+ mice express differentiation marker proteins. Detachment of podocytes into Bowman's space and tubular lumen was common. Double immunostaining showed that the nuclear WT1 signals were surrounded by podocalyxin, indicating that most podocytes remained mononuclear. Immunostaining revealed that a small number of podocytes were positive for γH2AX, a DNA damage marker, and cleaved lamin A, an apoptosis marker, with an average ratio of 0.26 and 0.39 per glomerulus, respectively, in SV40T+/rtTA+/Bcl2^+/+^ mice at P0 (Figure S7).

**FIGURE 3:**
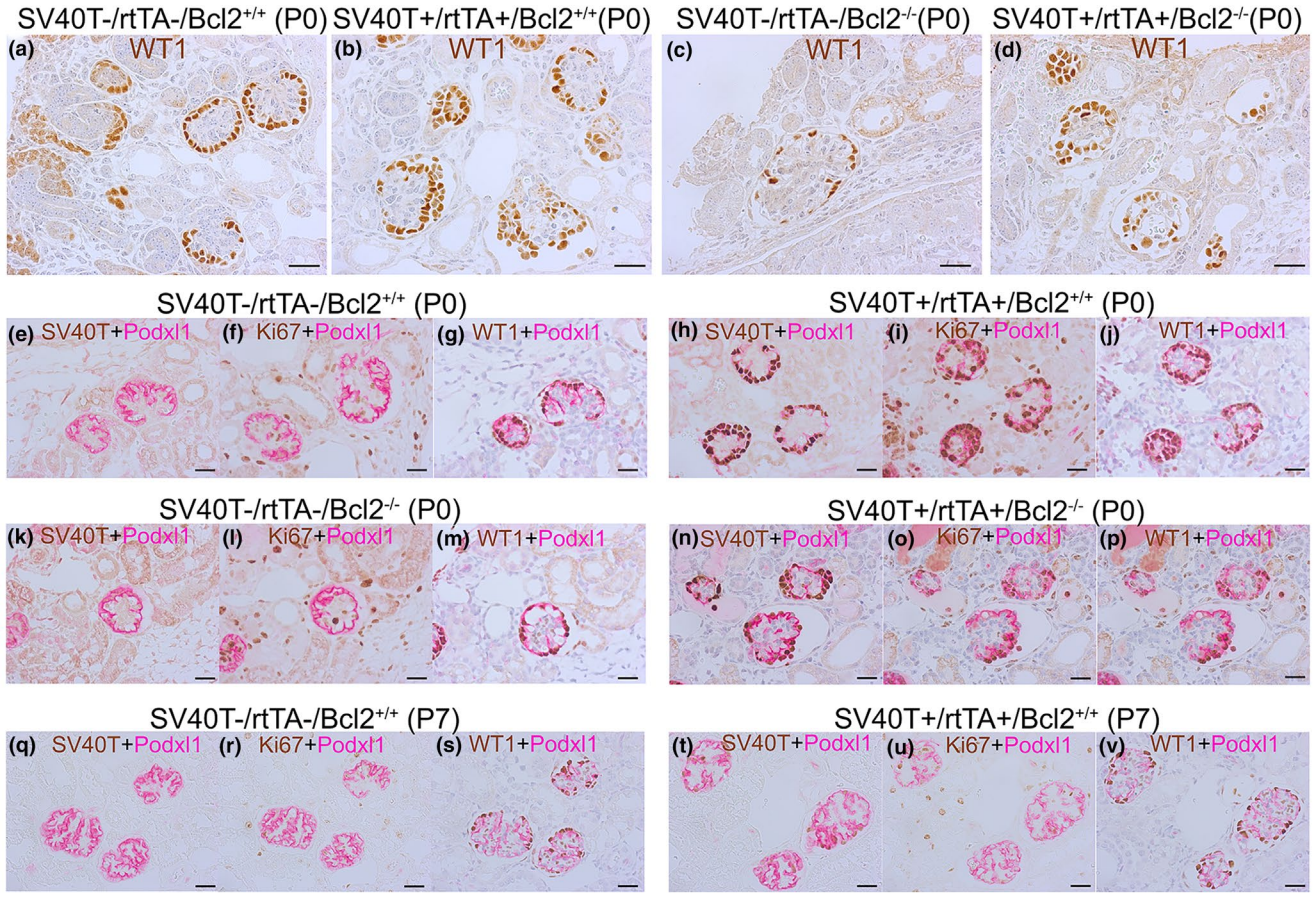
Podocyte proliferation in doxycycline‐treated newborn SV40T+/rtTA+ mice. Phenotypes at P0 (a–q): Regardless of Bcl2 genotype, SV40T+/rtTA+ mice exhibit a marked increase in podocyte number (b, d vs. a, c). Podocytes frequently form multilayers, detach from the glomerular tuft, and scatter within Bowman's capsule and tubular lumens. Double immunostaining demonstrates that most podocytes (identified by pink podocalyxin (Podxl1) staining) express SV40T (h, n), and many express Ki67 (i, o). Phenotypes at P7 (q–v): In SV40T+/rtTA+ mice, only a subset of podocytes express SV40T (t) and fewer podocytes are Ki67‐positive (u). Most podocytes form a monolayer (v), similar to the control mice (s). Panels e–g, h–j, k–m, n–p, q–s, and t–v show visual fields that are comparable to those of the adjacent sections. Bars represent 20 μm.

In SV40T+/rtTA+/Bcl2^+/+^ and control SV40T−/rtTA‐/Bcl2^+/+^ mice, podocyte number and glomerular tuft area were quantified (Figure 4). The number of WT1+ podocytes increased by an average of 54% in SV40T+/rtTA+/Bcl2^+/+^ mice, yet the tuft area did not significantly differ from that of the controls (Figure 4b). Although podocyte number and tuft area were correlated, even glomeruli with excessive podocytes did not exceed the size of control glomeruli (Figure 4c). These findings indicate that, despite clear proliferative activity, podocyte overproduction during late nephrogenesis does not enlarge the developing glomerular tuft.

**FIGURE 4:**
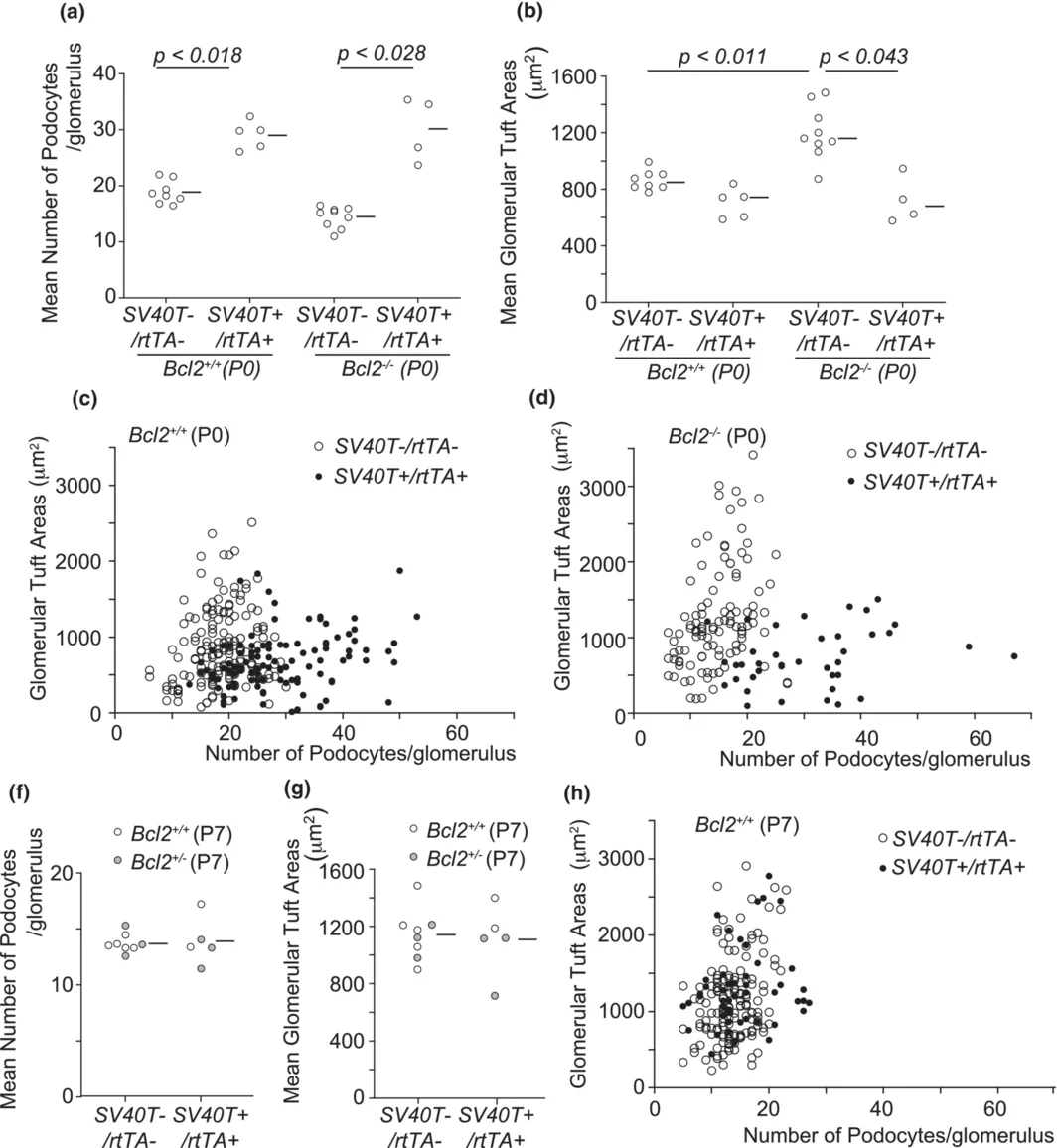
Quantitative analyses of podocyte number and glomerular tuft area in newborn mice. Scatterplots show mean podocyte number (a) and mean glomerular tuft area (b) at P0. In both Bcl2^+/+^ and Bcl2^−/−^ genotypes, podocyte number is higher in SV40T+/rtTA+ mice than in SV40T−/rtTA‐ mice (a). The tuft area does not differ significantly between SV40T+/rtTA+/Bcl2+/+ and SV40T−/rtTA‐/Bcl2^+/+^ mice. In Bcl2^−/−^ mice, the tuft area is larger in SV40T−/rtTA‐ than in SV40T+/rtTA+ mice; however, the tuft area is significantly smaller in SV40T+/rtTA+/Bcl2^−/−^ mice compared with SV40T−/rtTA‐/Bcl2^−/−^ mice (b). Individual glomerular data at P0 are plotted in 2D graphs (c for Bcl2^+/+^, d for Bcl2^−/−^). In both genotypes, the tuft areas of glomeruli with excessive podocyte numbers did not exceed those of the control glomeruli. At P7, both the podocyte number and the glomerular tuft area were comparable between SV40T+/rtTA+ and SV40T−/rtTA‐ mice (f and g). (h) 2D graph of podocyte number vs. tuft area at P7. The distributions of SV40T+/rtTA+ and control mice largely overlap. In contrast to SV40T+/rtTA+ mice at P0 (c), those at P7 (h) do not show glomeruli containing >30 podocytes.

### 3.4 Bcl2‐deficiency and embryonic podocyte proliferation

Independent of SV40T or rtTA genotype, all Bcl2^−/−^ mice exhibited severe renal hypoplasia. The glomeruli were markedly reduced in number, and most were located near the medulla. The cortical nephrogenic zone was absent (Figure 3c). In Bcl2^−/−^ mice lacking SV40T expression, podocytes occupied the glomerular surface and did not form multilayers. In contrast, SV40T+/rtTA+/Bcl2^−/−^ mice showed high SV40T and Ki67 expression in podocytes, with frequent multilayer formation and detachment from GBM (Figure 3d). WT1+ podocytes increased like that in SV40T+/rtTA+/Bcl2^+/+^ mice (Figure 4). Although the tuft area was larger in SV40T−/rtTA‐/Bcl2^−/−^ mice than in Bcl2^+/+^ controls, it was significantly reduced in SV40T+/rtTA+/Bcl2^−/−^ mice compared to SV40T−/rtTA‐/Bcl2^−/−^ littermates. Thus, even under conditions that normally induce compensatory glomerular enlargement, many podocytes did not remain attached to GBM.

### 3.5 Postnatal outcomes

Eight mothers were allowed to nurse pups under continuous doxycycline exposure, and 26 pups were analyzed at P7. No SV40T+/rtTA+/Bcl2^−/−^ mice were found among the surviving offspring. All glomeruli expressed podocalyxin. In SV40T+/rtTA+/Bcl2^+/+^ and SV40T+/rtTA+/Bcl2^+/−^ mice, podocytes displayed a normal arrangement without multilayering or detachment. At this stage, residual SV40T and Ki67 expression were slightly more frequent in outer cortical podocytes than in inner cortical podocytes (Figure S6). Approximately 20%–30% of inner‐cortical podocytes expressed SV40T, and only a small fraction was positive for Ki67 (Figure 3t–v). Data from Bcl2^+/+^ and Bcl2^+/−^ mice were pooled, and SV40T−/rtTA‐ and SV40T+/rtTA+ mice were compared. Podocyte number and tuft area did not differ significantly between the two groups (Figure 4f,g). Compared to P0, glomeruli containing excessively high podocyte numbers were no longer present at P7 (Figure 4h), suggesting that these surplus podocytes were eliminated postnatally. Taken together, these observations indicate that increased podocyte production during late embryogenesis is not sustained postnatally and does not lead to permanent glomerular enlargement.

## 4 DISCUSSION

In this study, we sought to increase the number of podocytes in vivo by generating a transgenic mouse line that enables podocyte‐specific, doxycycline‐inducible expression of SV40T. As anticipated, doxycycline administration induced cell cycle re‐entry in the podocytes of SV40T+/rtTA+ mice. However, in adult mice, SV40T expression and cell cycle re‐entry were inefficient, and cytokinesis often failed. Prolonged doxycycline exposure induced proteinuria and podocyte injury, possibly by dedifferentiation effects of SV40T, which in turn suppressed the Nphs2 promoter‐driven transgene expression. In addition, it was reported that expression of p16INK4a and p19INK4d is increased in aged podocytes (McKinzie et al., 2024), which reinforces their refractory nature to division. Moreover, protein leakage across the glomerular filtration barrier is presumably less problematic in the embryonic kidney, which has a low glomerular filtration rate. Therefore, we targeted embryonic development by administering doxycycline to pregnant dams with SV40T+/rtTA+ embryos.

Newborn SV40T+/rtTA+ mice exhibited marked podocyte proliferation, likely due to efficient SV40T expression, cell cycle re‐entry, and cytokinesis during development. Importantly, unlike adult mice with established FSGS, P0 podocytes maintained robust expression of key differentiation markers, including podocin, WT1, and podocalyxin, indicating that overt dedifferentiation had not occurred during this brief induction window. However, increased podocyte numbers did not result in glomerular enlargement. Instead, podocytes frequently became multilayered, detached from the GBM, and scattered in Bowman's space or tubular lumens. At present, it remains unclear whether this adhesive failure and loss of division orientation are intrinsic barriers to forcing complex, highly specialized podocytes to undergo mitosis, or whether they reflect SV40T‐specific off‐target effects on cell‐matrix adhesion and polarity. By P7, podocyte numbers in SV40T+/rtTA+ mice were normalized to those in the controls, suggesting that the supernumerary podocytes at P0 failed to stably adhere to the GBM and were subsequently lost. This indicates that expanding podocyte number by SV40T after the S‐shaped body stage cannot override the structural constraints that define glomerular size. Similar outcomes were observed in mice lacking Bcl2, which developed oligomeganephronia as a result of apoptosis of undifferentiated mesenchymal cells between E13 and E16 (Gassler et al., 1998; Nagata et al., 1996). Although SV40T+/rtTA+/Bcl2^−/−^ mice displayed increased podocyte numbers, many cells detached from the GBM. Despite glomerular hypertrophic pressure, the tuft area was reduced owing to the squeezing effect of podocytes.

Podocyte progenitors emerge at the S‐shaped body stage. In SV40T+/rtTA+ mice, SV40T expression in differentiating podocytes begins at the capillary loop stage due to Nphs2‐driven rtTA activity. Proliferation of podocyte progenitors at earlier stages is not stimulated. Our findings suggest that glomerular size is largely determined at or before the S‐shaped body stage, and increasing podocyte numbers after this developmental window cannot expand glomerular volumes.

In severe glomerular injury, mitotic podocytes often detach from GBM, a process commonly referred to as mitotic catastrophe (Lasagni et al., 2013). Originally, mitotic catastrophe was defined as an oncosuppressive mechanism that eliminates cells with mitotic defects arising from spindle checkpoint dysregulation, excessive DNA damage without an adequate G2 checkpoint, or premature mitotic exit. This process typically results in cell death during mitosis or the subsequent G1 phase (Sazonova et al., 2021; Vitale et al., 2011). In podocyte research, the term encompasses the detachment of mitotic podocytes from GBM (Lasagni et al., 2013). Forced cell cycle progression in podocytes may promote mitotic error and subsequent mitotic death. In SV40T+/rtTA+ mice at P0, only a small number of podocytes were positive for γH2AX and cleaved lamin A (Figure S7), indicating DNA damage and apoptosis in a subset of post‐mitotic cells. The scarcity of these markers in SV40T‐expressing podocytes suggests that adhesive failure due to mitotic rounding rather than programmed cell death is the dominant driver of podocyte loss.

During mitosis, most epithelial cells, including tubular epithelial cells, round up and temporarily reduce integrin‐mediated adhesion to the basement membrane, but re‐establish adhesion after division (Huber et al., 2023). In contrast, podocytes in adult mice are exposed to high filtration pressures, which may promote detachment or irreversible architectural defects during mitosis. Although embryonic glomeruli are subject to low filtration pressure, newborn SV40T+/rtTA+ mice, including those delivered via cesarean section, also demonstrated frequent podocyte detachment, indicating that high filtration pressure is not the sole determinant. Rather, the highly specialized and intricate architecture of podocytes renders them intrinsically vulnerable to the structural reorganization required for cell division.

Proliferating podocytes often formed multilayers in newborn SV40T+/rtTA+ mice. In contrast, renal tubular epithelial cells strictly keep a planar division (division perpendicular to the apical–basal axis), maintaining a monolayer architecture even during cyst formation (Gao et al., 2017; Simons & Walz, 2006). Podocytes may lack such stringent control of division orientation, thereby contributing to abnormal multilayering and detachment from GBM. Another possibility is that supernumerary podocytes are eliminated by a mechanism called “cell competition.” Cell competition is a quality‐control mechanism in which neighboring cells compare their relative fitness, leading more fit “winner” cells to actively eliminate less fit “loser” cells and thereby maintain tissue integrity and homeostasis (Hogan et al., 2009; Kajita et al., 2010). In epithelial cells, this process can act as an intrinsic tumor‐suppressive mechanism. In our model, SV40T‐induced podocytes likely represent “unfit” cells within the developing glomerular architecture. An endogenous quality control program may recognize these supernumerary cells as “losers” and trigger their elimination. This suggests that podocyte number is not determined solely by initial production but is regulated by a rigorous “occupancy management” system that preserves the glomerular microarchitecture.

In summary, our findings demonstrate that forcing committed podocytes to re‐enter the cell cycle via SV40T expression from the capillary loop stage onward fails to expand permanent glomerular volume, resulting instead in detachment and postnatal loss of supernumerary cells. These results indicate that glomerular size and structural capacity are strictly constrained during early nephrogenesis (at or before the S‐shaped body stage). Overcoming these biological constraints for potential podocyte expansion would likely require strategies that act at earlier progenitor stages or control cell division orientation and cell‐matrix adhesion to allow stable integration into the glomerular tuft.

## AUTHOR CONTRIBUTIONS

**Taiji Matsusaka:** Conceptualization; data curation; formal analysis; funding acquisition; investigation; methodology; project administration; resources. **Koichiro Ichimura:** Supervision; validation. **Takayuki Miyaki:** Supervision; validation. **Masahiro Koizumi:** Conceptualization; formal analysis; investigation; methodology.

## FUNDING INFORMATION

TM received grants (Grant‐in‐Aid for Scientific Research, 14657256, 16109005, 18209030, 23249047, and 21H02936) from the Japan Society for the Promotion of Science (https://www.jsps.go.jp/english/e‐grants/index.html). The funders had no role in the study design, data collection and analysis, decision to publish, or manuscript preparation.

## CONFLICT OF INTEREST STATEMENT

The authors declare no competing interests.

## DECLARATION OF GENERATIVE AI AND AI‐ASSISTED TECHNOLOGIES

Generative AIs (ChatGPT‐4, Gemini 3 Flash, and Gemini 3.5 Flash) were used to correct grammar and spelling in the whole manuscript and reorganize the Discussion section for clarity and flow. All AI‐assisted text was reviewed and revised by the authors to ensure accuracy and clarity of meaning.

## ETHICS STATEMENT

All animal experiments in this study were approved by the Animal Experimentation Committee of Tokai University School of Medicine (approval numbers: 122034, 133015, 144028, and 155027). The procedures were conducted in strict accordance with the Guide for the Care and Use of Laboratory Animals of the U.S. National Institutes of Health (NIH) and adhered to the ARRIVE guidelines (Animal Research: Reporting of In Vivo Experiments). All efforts were made to minimize animal suffering, and mice were humanely euthanized under isoflurane inhalation anesthesia.

## Supporting information


Figure S1.

Figure S2.

Figure S3.

Figure S4.

Figure S5.

Figure S6.

Figure S7.

Table S1.

Table S2.

Table S3.

Table S4.

## Data Availability

All data generated or analyzed during this study are included in this published article and Figures [Link], [Link] and Tables [Link], [Link]. Further enquiries can be directed to the corresponding author.
