# Supplementary material for: Increase in podocyte number induced by SV40T antigen during the fetal stage does not expand glomerular size in mice
Source: Physiol Rep. 2026 Oct 2;14(19):e71114. doi: 10.14814/phy2.71114 (PMC13633475; doi:10.14814/phy2.71114)
Supplement: Supplementary file 1 [file PHY2-14-e71114-s001.pdf]

S1 Fig.

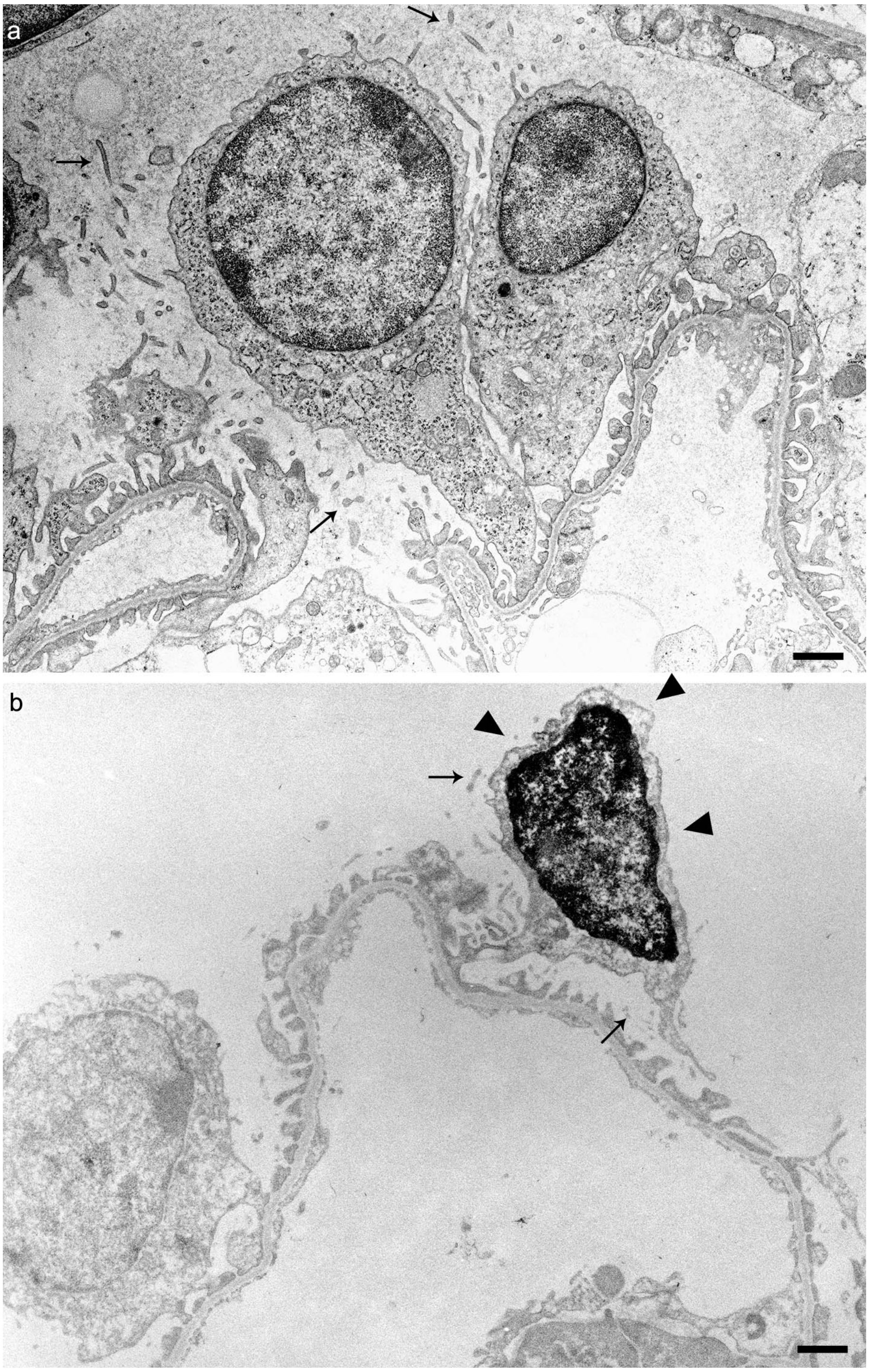

Ultrastructure of podocytes from SV40T/rtTA mice treated with doxycycline

(a) Conventional electron microscopy of SV40T+/rtTA+ mice treated with doxycycline for 7 days reveals two adjacent podocytes exhibiting microvillous transformation (arrows), while foot processes and other structures remain normal.

(b) BrdU immuno-EM: mice were treated with doxycycline for 4 days with continuous BrdU infusion, followed by immediate analysis. A BrdU-incorporated podocyte (arrowheads) displays normal foot processes and mild microvilous transformation (arrows).

Bars represent 1 μm.
