# Supplementary material for: Increase in podocyte number induced by SV40T antigen during the fetal stage does not expand glomerular size in mice
Source: Physiol Rep. 2026 Oct 2;14(19):e71114. doi: 10.14814/phy2.71114 (PMC13633475; doi:10.14814/phy2.71114)
Supplement: Supplementary file 2 [file PHY2-14-e71114-s004.pdf]

S5 Fig.

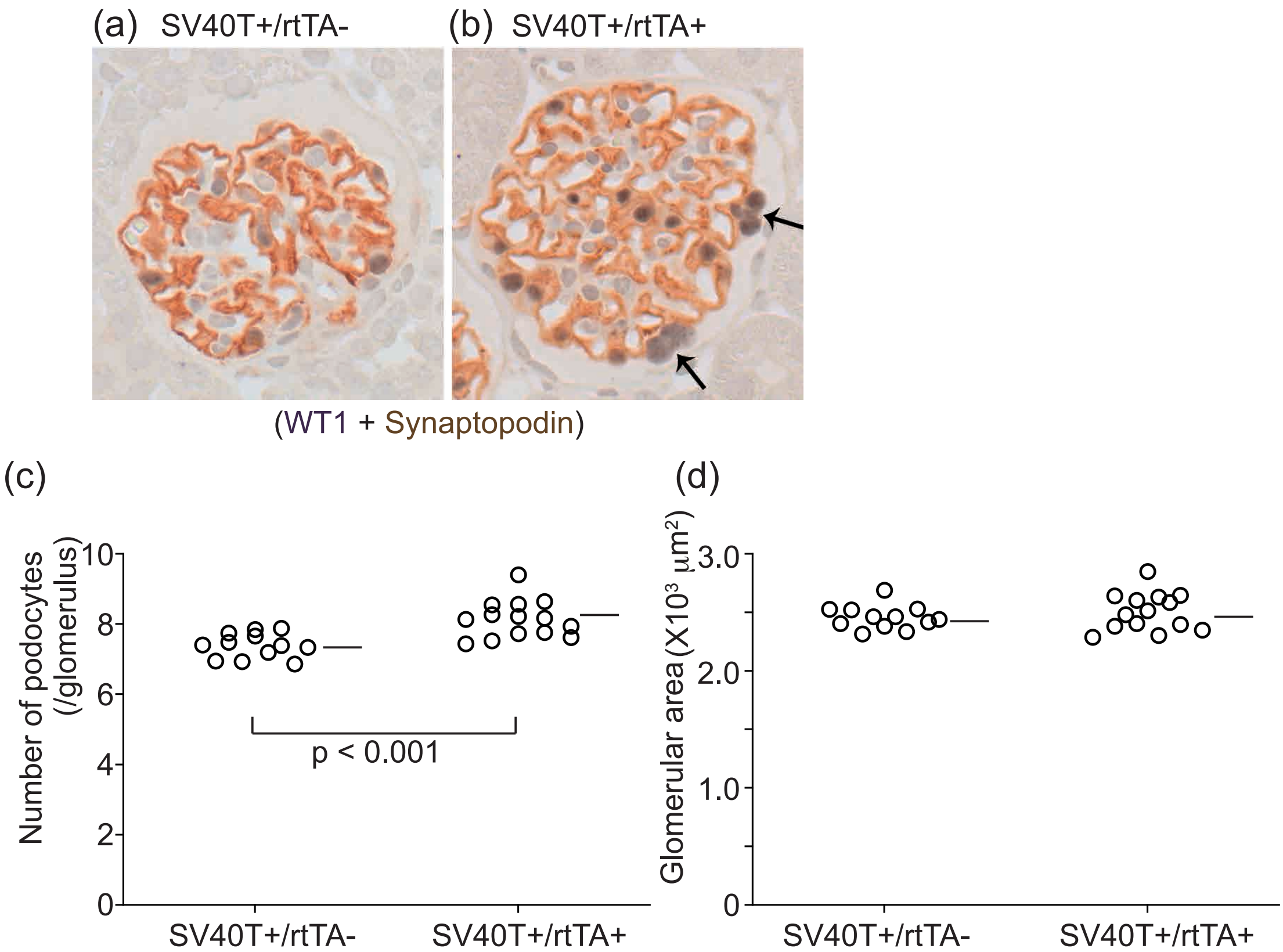

Podocytes after multiple intermittent doxycycline treatments.

Fourteen SV40T+/rtTA+ mice and 12 age- and sex-matched SV40T+/rtTA- controls were treated with doxycycline and losartan for 4 days, followed by losartan alone for 10 days. This cycle was repeated six times. Kidney sections were double-stained for WT1 (gray) and synaptopodin (brown) (a, b), and podocytes were counted. Non-podocytes with faint WT1 staining (e.g., arrowhead in a) lacked synaptopodin expression and were frequently located on the luminal side. In SV40T+/rtTA+ mice, 6.4 ± 1.3% of podocytes were multinuclear (arrows in b). Multinuclear podocytes were counted as single podocytes. Podocyte number per glomerulus was significantly higher in SV40T+/rtTA+ mice than in controls (c). Glomerular size did not differ between groups (d).
