# Supplementary material for: Increase in podocyte number induced by SV40T antigen during the fetal stage does not expand glomerular size in mice
Source: Physiol Rep. 2026 Oct 2;14(19):e71114. doi: 10.14814/phy2.71114 (PMC13633475; doi:10.14814/phy2.71114)
Supplement: Supplementary file 3 [file PHY2-14-e71114-s005.pdf]

S6 Figure

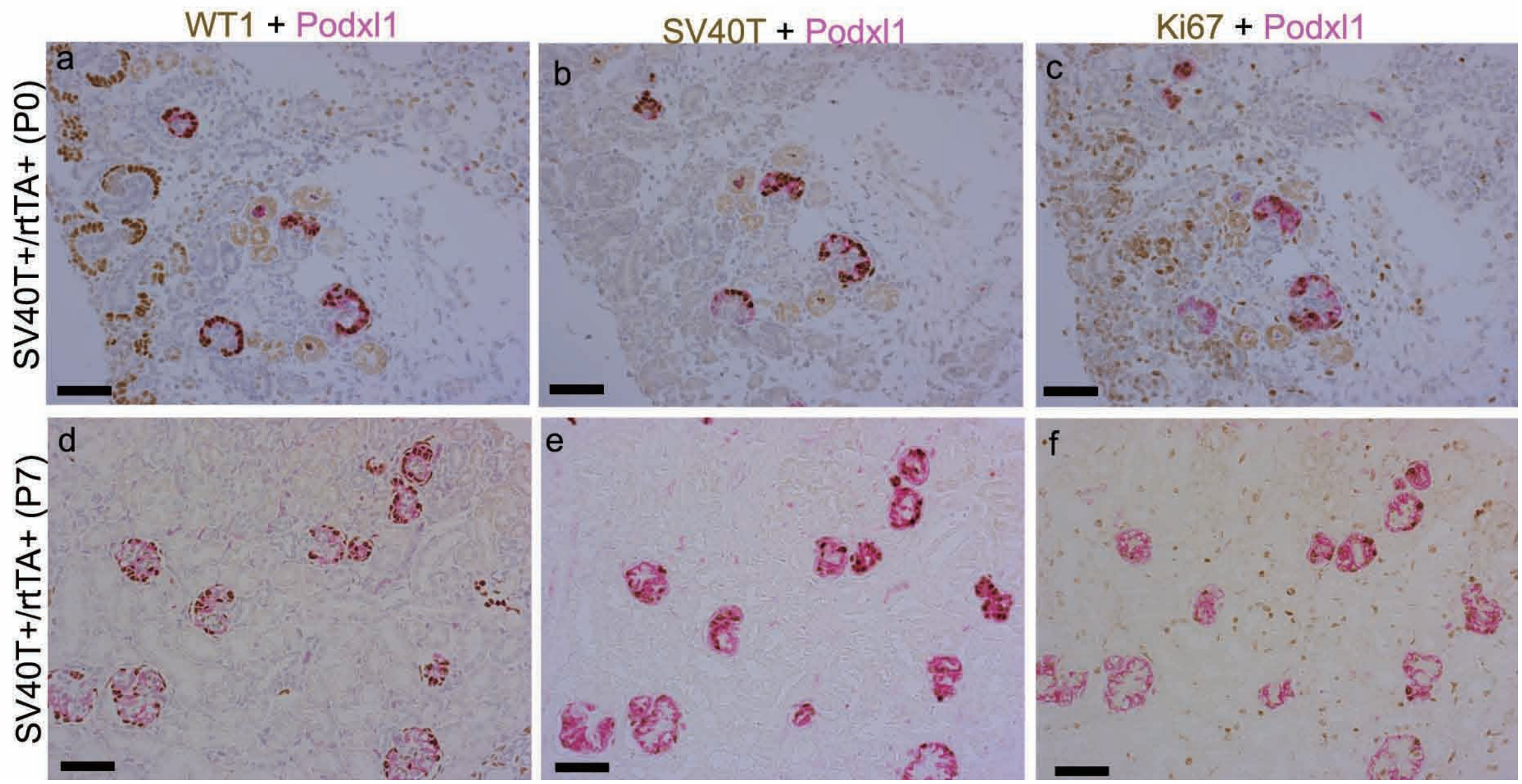

Comparison of glomeruli between the inner and outer cortex of newborn SV40T+/rtTA+ mice. Double immunostaining for podocalyxin (Podxl1, pink) and WT1 (a, d), SV40T (b, e), or Ki67 (c, f).

Phenotypes at P0 (a–c): most glomerular precursors in the outer renal cortex had not yet expressed podocalyxin or SV40T. In rare and slightly more mature superficial glomeruli, podocalyxin was expressed, but SV40T expression was sporadic, and podocytes did not express Ki67 nor did they form multilayers. Conversely, inner cortical glomeruli displayed strong SV40T/Ki67 expression and prominent multilayering.

Phenotypes at P7 (d–f): all glomeruli expressed podocalyxin. At this stage, residual SV40T and Ki67 expression were slightly more frequent in outer cortical podocytes than in inner cortical podocytes.

Scale bars: 50 μm in a–c; 200 μm in d–f.
