# Supplementary material for: Increase in podocyte number induced by SV40T antigen during the fetal stage does not expand glomerular size in mice
Source: Physiol Rep. 2026 Oct 2;14(19):e71114. doi: 10.14814/phy2.71114 (PMC13633475; doi:10.14814/phy2.71114)
Supplement: Supplementary file 4 [file PHY2-14-e71114-s007.pdf]

**S3 Fig.**

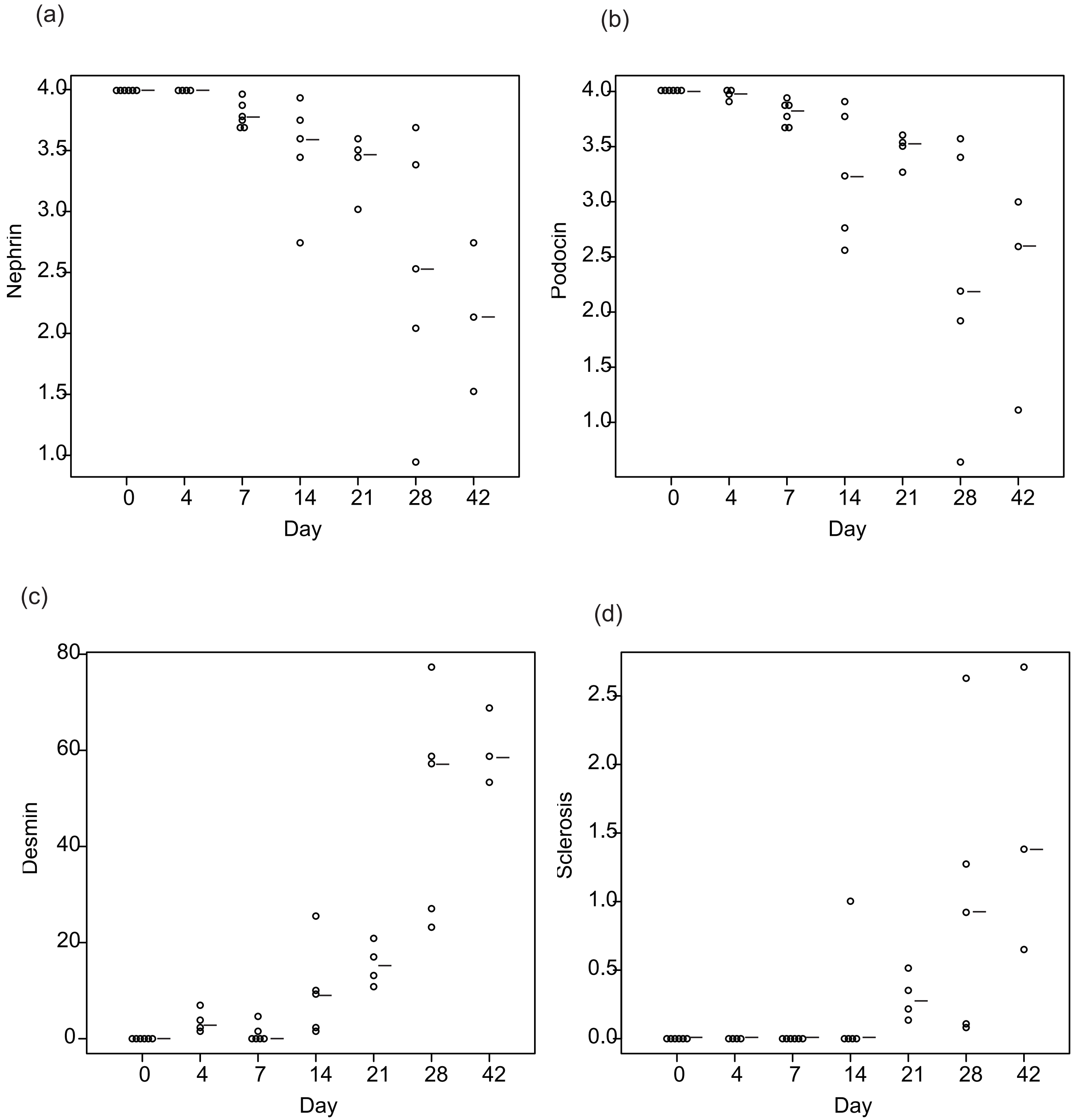


**Podocyte injury in SV40T+/rtTA+ mice with long-term doxycycline treatment**

SV40T+/rtTA+ mice were continuously treated with doxycycline for the indicated durations. Nephrin, podocin, and desmin staining, along with glomerulosclerosis severity, were semi-quantified. Bars indicate medians. Corrected p-values (Kruskal–Wallis rank-sum tests) were all $<0.001$.
