# Supplementary material for: Increase in podocyte number induced by SV40T antigen during the fetal stage does not expand glomerular size in mice
Source: Physiol Rep. 2026 Oct 2;14(19):e71114. doi: 10.14814/phy2.71114 (PMC13633475; doi:10.14814/phy2.71114)
Supplement: Supplementary file 5 [file PHY2-14-e71114-s008.pdf]

S7 Figure

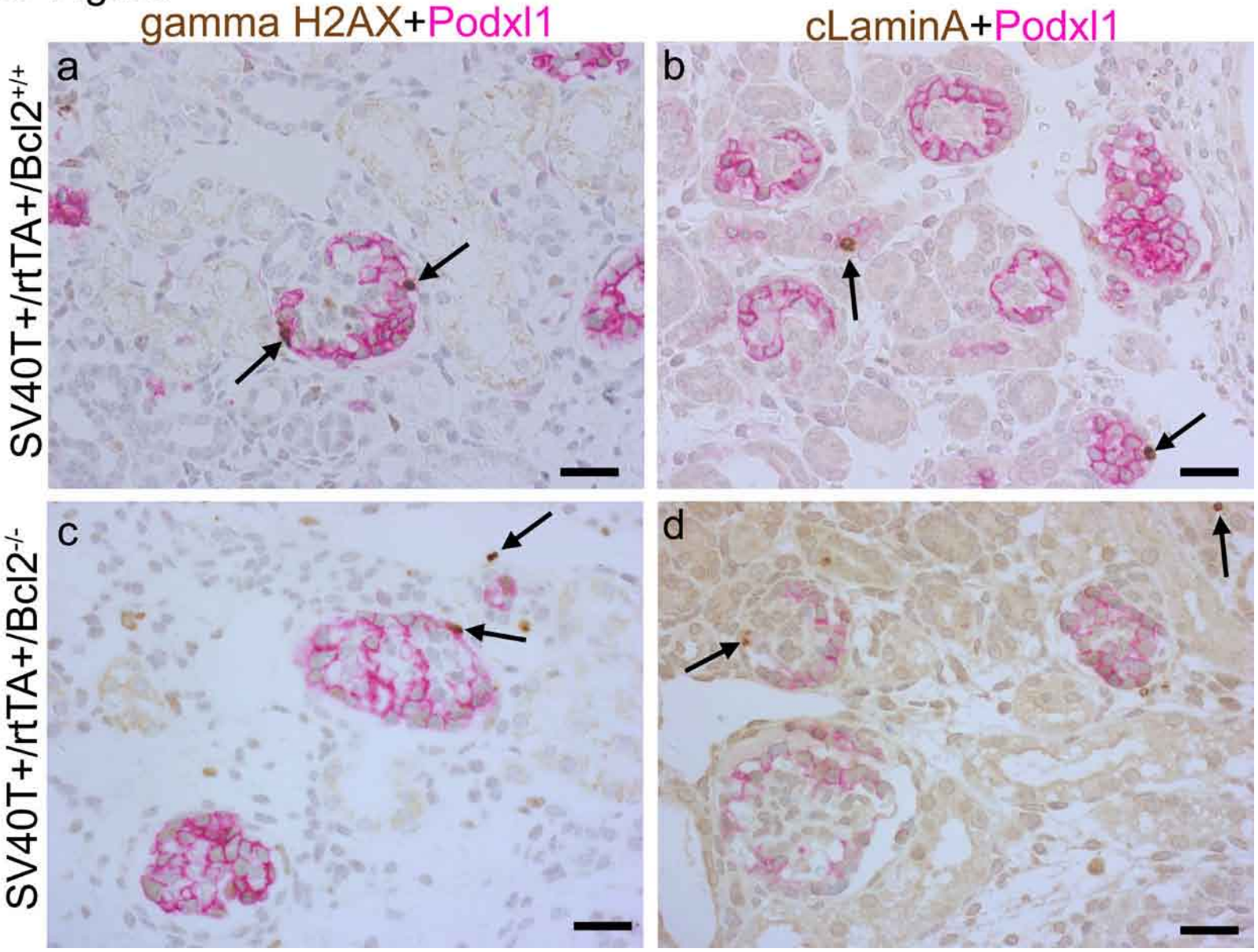

DNA damage and apoptosis in podocytes of SV40T+/rtTA+ mice.

DNA damage and apoptosis in podocytes of SV40T+/rtTA+ mice. Representative pictures of SV40T+/rtTA+/Bcl2+/+ (a and b) and SV40T+/rtTA+/Bcl2-/- (c and d) mouse kidneys at P0 stained for gamma H2AX (arrows) and podocalyxin (Podxl1) (a and c) and cleaved lamin A (cLaminA; arrows) and Podxl1(b and d).

Scale bar: 20 µm.

The average number of gamma H2AX and cLaminA-positive cells per glomerulus was 0.26 and 0.39 in SV40T+/rtTA+/Bcl2+/+ mice, and 0.15 and 0.15 in SV40T+/rtTA+/Bcl2-/- mice, respectively.
