# Supplementary material for: Increase in podocyte number induced by SV40T antigen during the fetal stage does not expand glomerular size in mice
Source: Physiol Rep. 2026 Oct 2;14(19):e71114. doi: 10.14814/phy2.71114 (PMC13633475; doi:10.14814/phy2.71114)
Supplement: Supplementary file 6 [file PHY2-14-e71114-s010.pdf]

S2 Figure.

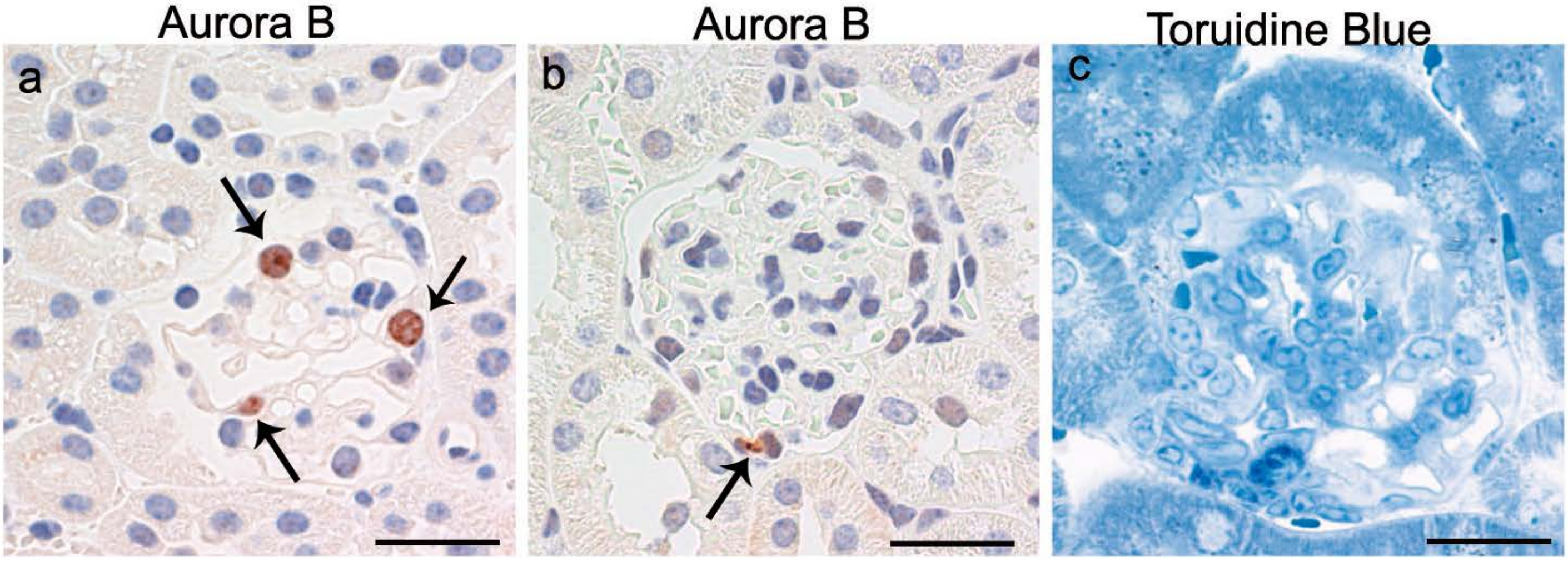

Cell-cycle re-entry of podocytes in doxycycline-treated SV40T+/rtTA+ mice
(a, b) Aurora B kinase expression in podocytes. Aurora B is frequently detected in podocytes of doxycycline-treated SV40T+/rtTA+ mice. Most Aurora B staining localizes to the nucleus (a, arrows), while 7.3% localizes outside the nucleus (b, arrow).(c) Podocyte mitosis. Mitotic figures (arrow) are occasionally observed in podocytes of doxycycline-treated SV40T+/rtTA+ mice.
Bars represent 20 µm.
