# Supplementary material for: Increase in podocyte number induced by SV40T antigen during the fetal stage does not expand glomerular size in mice
Source: Physiol Rep. 2026 Oct 2;14(19):e71114. doi: 10.14814/phy2.71114 (PMC13633475; doi:10.14814/phy2.71114)
Supplement: Supplementary file 7 [file PHY2-14-e71114-s011.pdf]

S4 Fig.

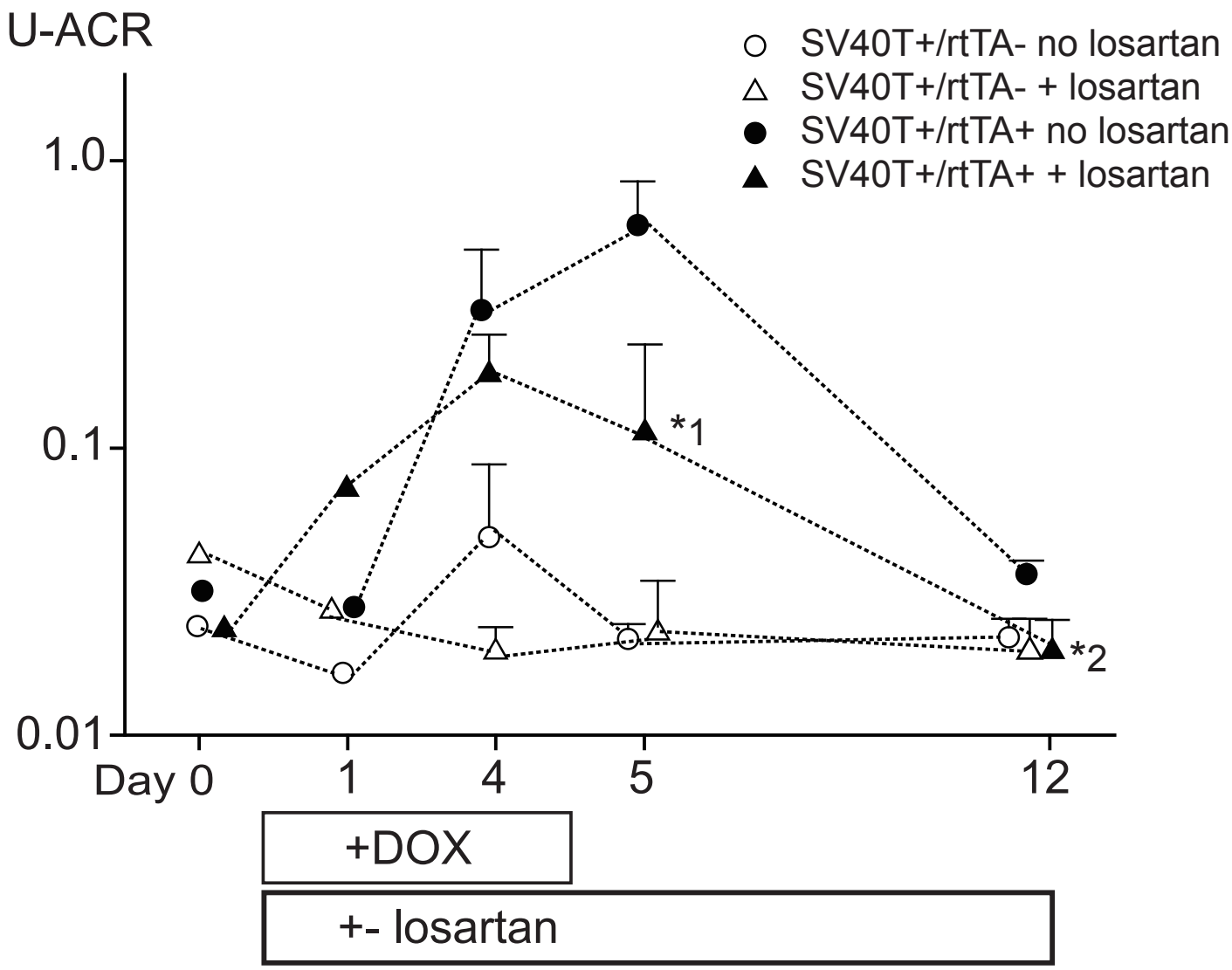

Albuminuria in SV40T+/rtTA+ mice treated with doxycycline.

Female SV40T+/rtTA+ and control SV40T+/rtTA- mice (n = 6 per group) were treated with doxycycline for 4 days, with or without continuous losartan. Urinary albumin/creatinine ratios (U-ACR) were measured from 24-hour urine samples. Geometric means and SEs are shown.

Microalbuminuria induced by short-term doxycycline treatment is reversible upon DOX withdrawal and was attenuated by concurrent losartan..

*1) $p < 0.001$ vs. SV40T+/rtTA+ without losartan; *2) $p = 0.040$ vs. SV40T+/rtTA+ without losartan.
