# Supplementary material for: Increase in podocyte number induced by SV40T antigen during the fetal stage does not expand glomerular size in mice
Source: Physiol Rep. 2026 Oct 2;14(19):e71114. doi: 10.14814/phy2.71114 (PMC13633475; doi:10.14814/phy2.71114)
Supplement: Supplementary file 8 [file PHY2-14-e71114-s009.docx]

**S4 Table.** Prevalence of SV40T (+) and Ki67 (+) podocytes in adult *rtTA+/SV40T+* mice

| Duration of doxycycline | Number of mice studied | SV40T (+) podocytes (%)  (mean ± SE) | Ki67 (+) podocyte (%)  (mean ± SE) |
| --- | --- | --- | --- |
| 0 days | 8 | 0.0 ± 0.0 | 0.0 ± 0.0 |
| 4 days | 7 | 23.7 ± 2.5 | 9.3 ± 1.7 |
| 7 days | 6 | 30.3 ± 1.3 | 17.7 ± 1.0 |
| 14 days | 4 | 20.0 ± 0.8 | 12.4 ± 1.5 |

The number of Ki67+ podocytes was determined by double staining for Ki67 and synaptopodin. The number of SV40T (+) or Ki67(+) podocytes is expressed as a percentage of the total number of podocytes that were counted in the adjacent sections stained for WT1.

None of the mice with the other genotypes showed SV40T or Ki67 following treatment with doxycycline.
